# Supplementary material for: A dynamic rhizosphere interplay between tree roots and soil bacteria under drought stress
Source: eLife. 2022 Jul 20;11:e79679. doi: 10.7554/eLife.79679 (PMC9385208; doi:10.7554/eLife.79679)
Supplement: Figure 7—source data 1. [file elife-79679-fig7-data1.pdf]

TOC-Assimilation Statistics

```
library(tidyverse)
```

```
## -- Attaching packages --------------------------------------- tidyverse 1.3.1 -
-
```

```
## v ggplot2 3.3.5     v purrr   0.3.4
## v tibble  3.1.5     v dplyr   1.0.7
## v tidyr   1.1.4     v stringr 1.4.0
## v readr   2.0.2     v forcats 0.5.1
```

```
## -- Conflicts ------------------------------------------ tidyverse_conflicts() -
-
## x dplyr::filter() masks stats::filter()
## x dplyr::lag()    masks stats::lag()
```

```
library(readxl)
library(emmeans)
setwd("C:/Dropbox (Weizmann Institute)/Labs/Klein/Yaara/Paper/")
dat <- read_xlsx("For TOC covariate.xlsx", sheet="combined")
dat$treatment1 <- as.factor(dat$treatment1)
dat$treatment2 <- as.factor(dat$treatment2)
```

Pre Drought

```
ggplot(data=dat, aes(x=assim1, y=toc1)) +
  geom_point(aes(color=treatment1, shape=treatment2))
```

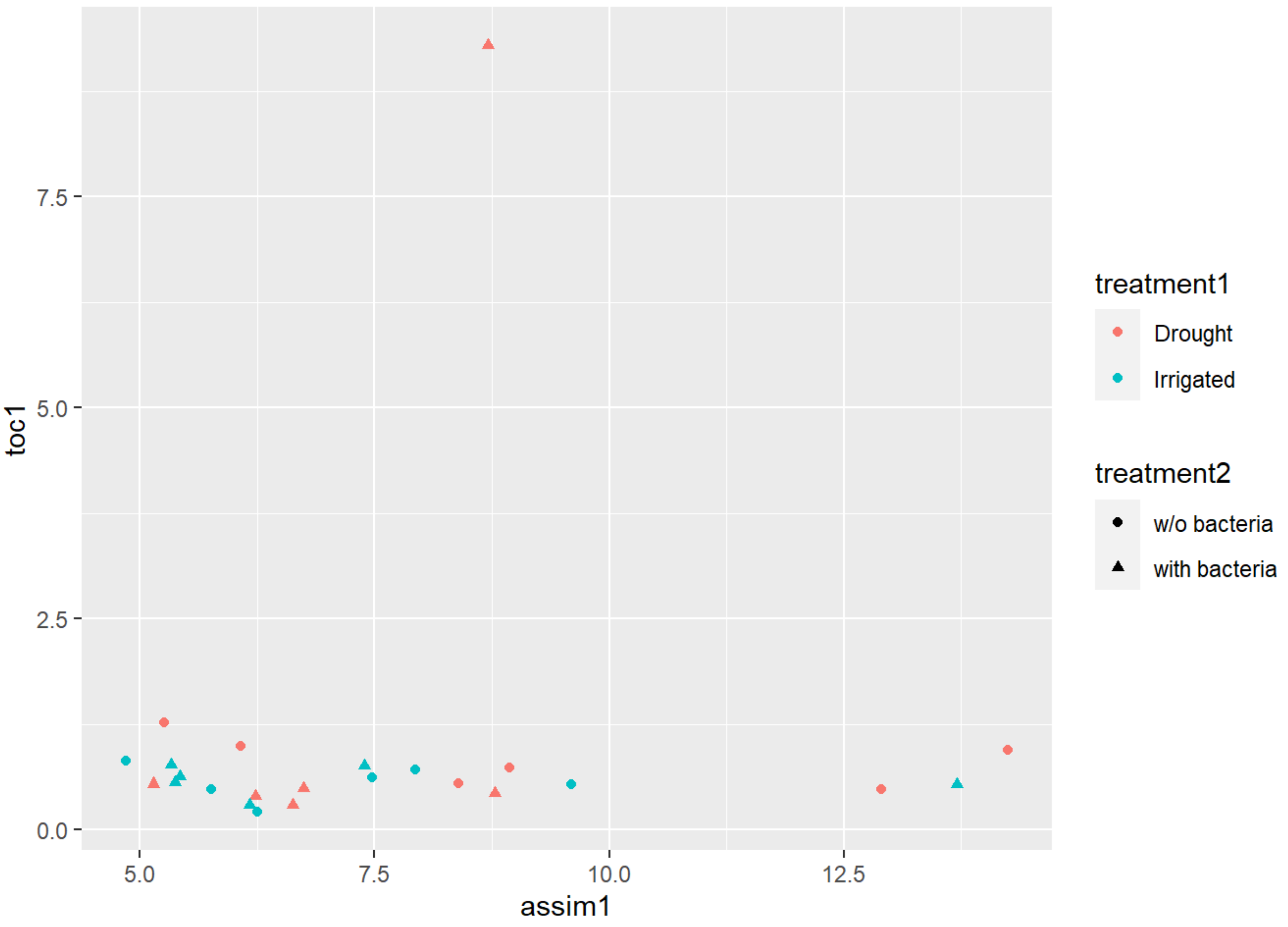


```
m1 <- lm(toc1 ~ treatment1 * treatment2 + assim1, data=dat)
summary(m1)
```

```
##
## Call:
## lm(formula = toc1 ~ treatment1 * treatment2 + assim1, data = dat)
##
## Residuals:
##     Min      1Q  Median      3Q     Max
## -1.6167 -0.5897 -0.1348  0.1618  7.2506
##
## Coefficients:
##                                             Estimate Std. Error t value
## (Intercept)                                  0.08605    1.62844   0.053
## treatment1Irrigated                         -0.08134    1.12989  -0.072
## treatment2with bacteria                      1.25943    1.12692   1.118
## assim1                                       0.08011    0.15497   0.517
## treatment1Irrigated:treatment2with bacteria -1.24782    1.56389  -0.798
##                                             Pr(>|t|)
## (Intercept)                                    0.958
## treatment1Irrigated                            0.943
## treatment2with bacteria                        0.278
## assim1                                         0.611
## treatment1Irrigated:treatment2with bacteria    0.435
##
## Residual standard error: 1.855 on 19 degrees of freedom
## Multiple R-squared:  0.111,  Adjusted R-squared:  -0.07618
## F-statistic: 0.593 on 4 and 19 DF,  p-value: 0.6719
```

Assimilation by irrigation

```
ggplot(data=dat, aes(x=treatment1, y=assim1)) +
  geom_boxplot() +
  geom_point()
```

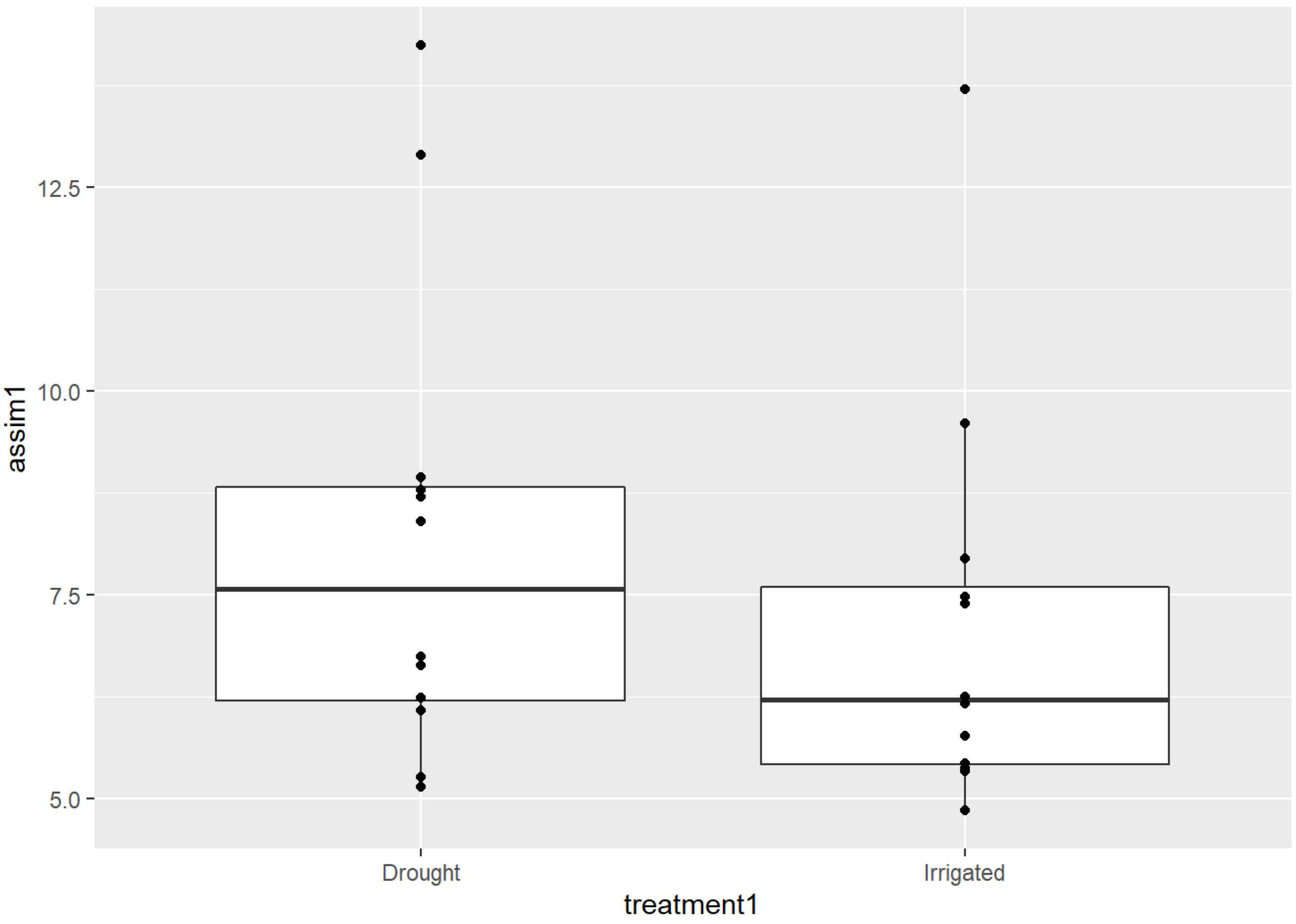


```
t.test(assim1 ~ treatment1, data=dat)
```

```
##
##  Welch Two Sample t-test
##
## data:  assim1 by treatment1
## t = 0.97249, df = 21.562, p-value = 0.3416
## alternative hypothesis: true difference in means between group Drought and grou
p Irrigated is not equal to 0
## 95 percent confidence interval:
##  -1.210864  3.344429
## sample estimates:
##   mean in group Drought mean in group Irrigated
##                8.170932                7.104149
```

Drought peirod

```
ggplot(data=dat, aes(x=assim2, y=toc2)) +
  geom_point(aes(color=treatment1, shape=treatment2))
```

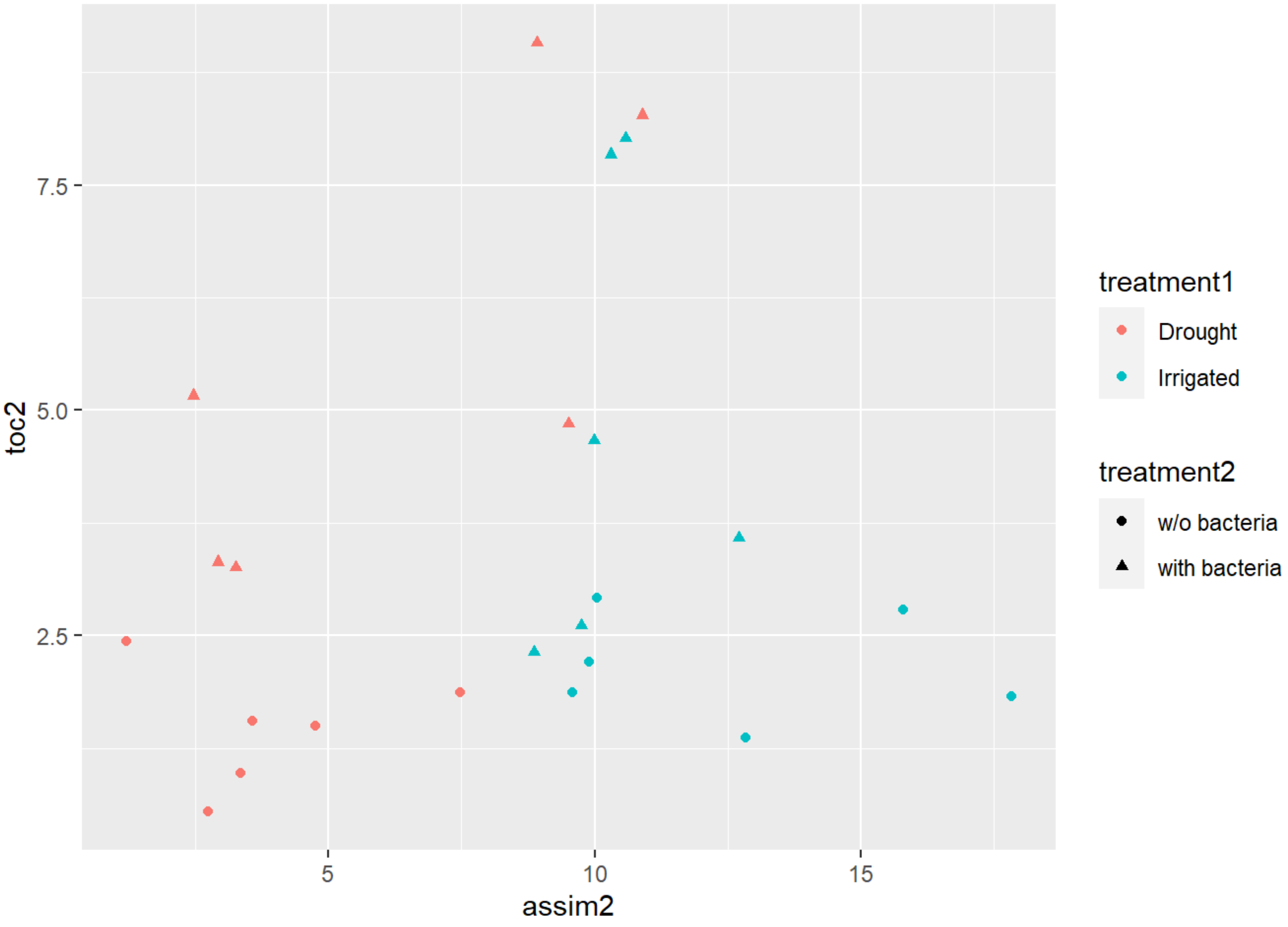


```
m2 <- lm(toc2 ~ treatment1 * treatment2 + assim2, data=dat)
summary(m2)
```

```
##
## Call:
## lm(formula = toc2 ~ treatment1 * treatment2 + assim2, data = dat)
##
## Residuals:
##     Min      1Q  Median      3Q     Max
## -2.1821 -1.5127 -0.1372  0.8410  3.1351
##
## Coefficients:
##                                             Estimate Std. Error t value
## (Intercept)                                   0.6074     0.8877   0.684
## treatment1Irrigated                          -1.3119     1.5711  -0.835
## treatment2with bacteria                       3.6161     1.0665   3.391
## assim2                                        0.2264     0.1364   1.659
## treatment1Irrigated:treatment2with bacteria  -0.4183     1.5722  -0.266
##                                             Pr(>|t|)
## (Intercept)                                  0.50205
## treatment1Irrigated                          0.41409
## treatment2with bacteria                      0.00307 **
## assim2                                       0.11344
## treatment1Irrigated:treatment2with bacteria  0.79308
## ---
## Signif. codes:  0 '***' 0.001 '**' 0.01 '*' 0.05 '.' 0.1 ' ' 1
##
## Residual standard error: 1.752 on 19 degrees of freedom
## Multiple R-squared:  0.5852, Adjusted R-squared:  0.4979
## F-statistic: 6.702 on 4 and 19 DF,  p-value: 0.001535
```

Assimilation by irrigation

```
ggplot(data=dat, aes(x=treatment1, y=assim2)) +
  geom_boxplot() +
  geom_point()
```

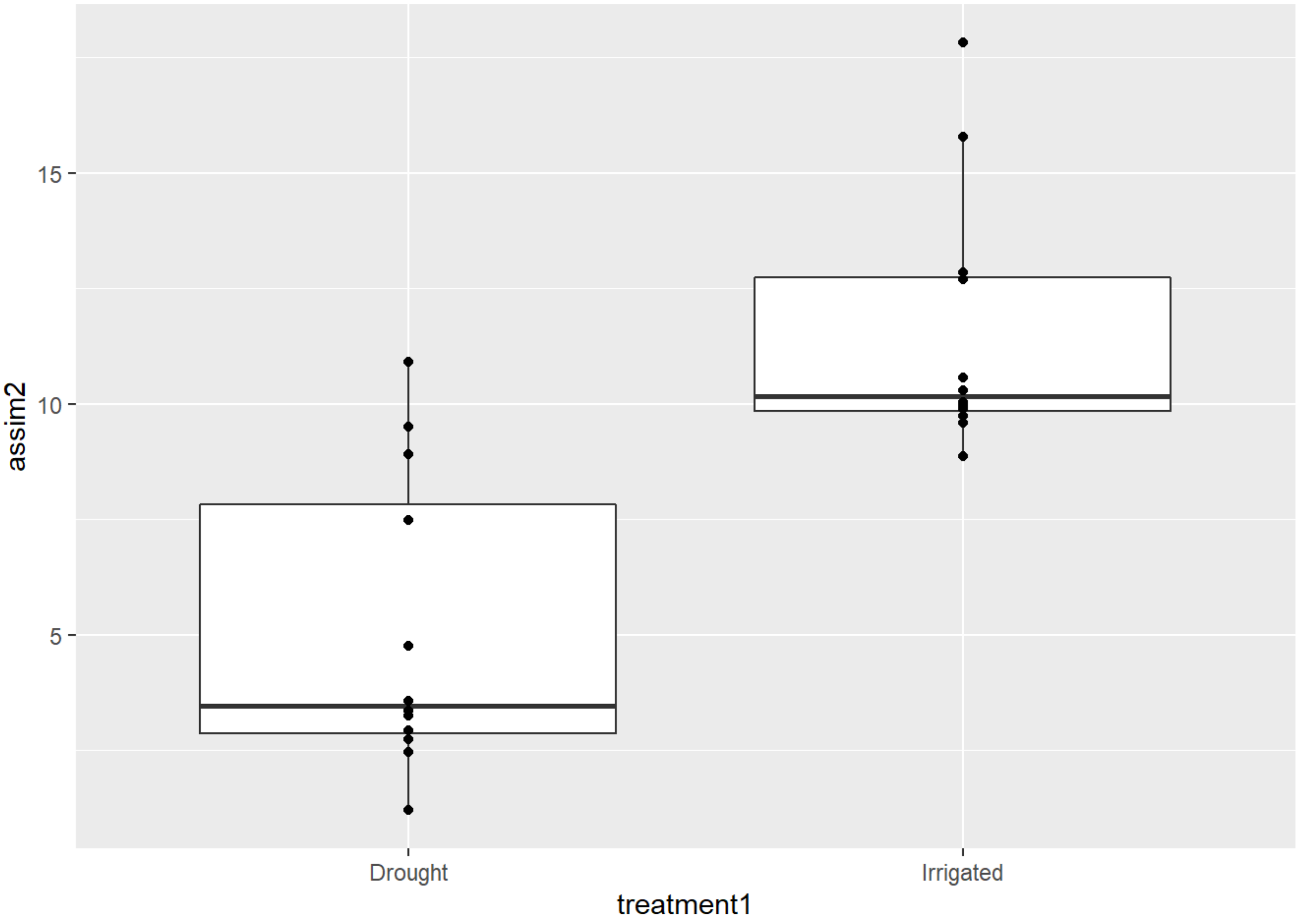


```
t.test(assim2 ~ treatment1, data=dat)
```

```
##
##  Welch Two Sample t-test
##
## data:  assim2 by treatment1
## t = -5.2272, df = 21.515, p-value = 3.259e-05
## alternative hypothesis: true difference in means between group Drought and grou
p Irrigated is not equal to 0
## 95 percent confidence interval:
##  -8.972579 -3.870485
## sample estimates:
##   mean in group Drought mean in group Irrigated
##                5.090818               11.512350
```

Re-Irrigated

```
ggplot(data=dat, aes(x=assim3, y=toc3)) +
  geom_point(aes(color=treatment1, shape=treatment2))
```

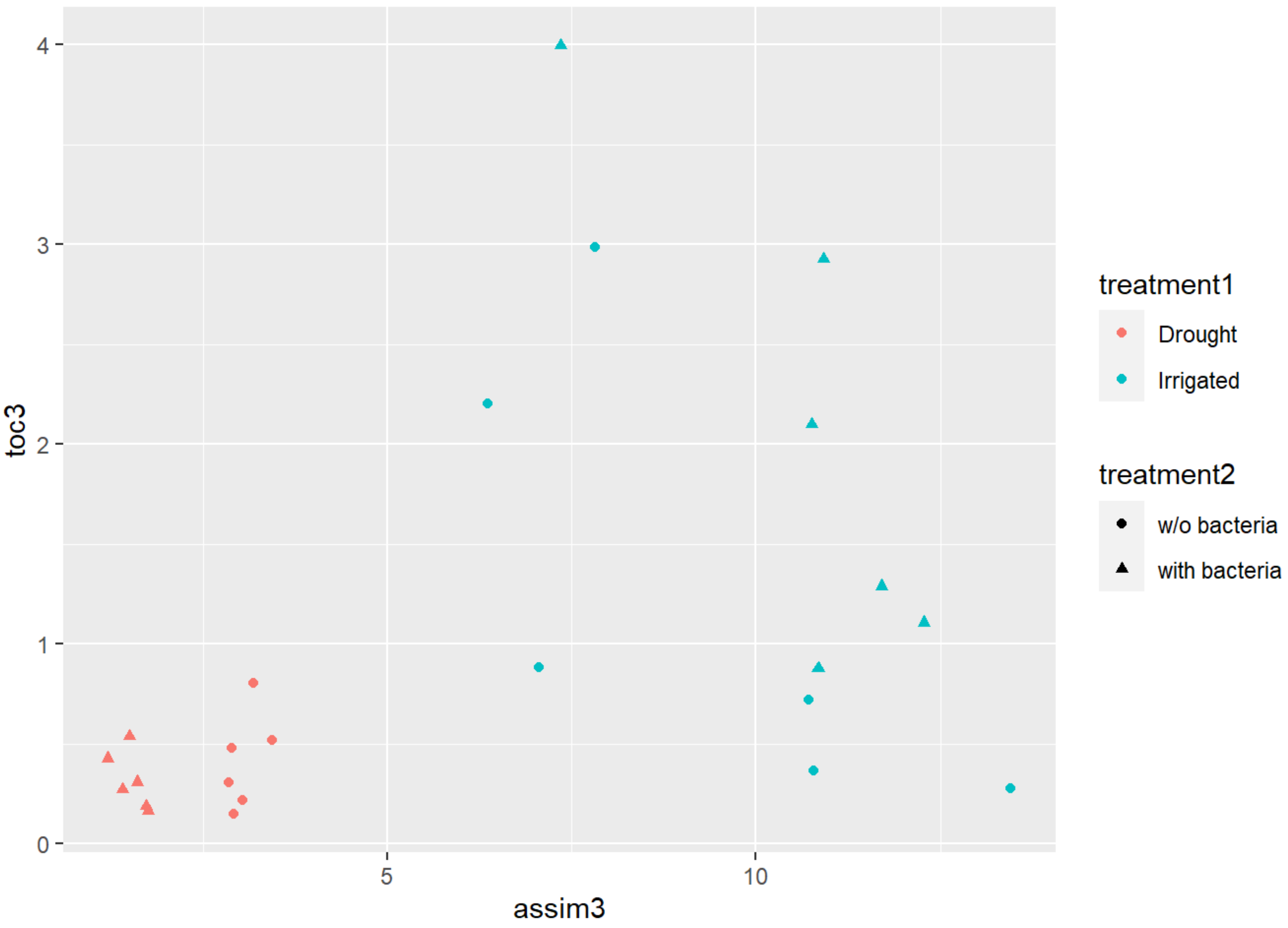


```
m3 <- lm(toc3 ~ treatment1 * treatment2 + assim3, data=dat)
summary(m3)
```

```
##
## Call:
## lm(formula = toc3 ~ treatment1 * treatment2 + assim3, data = dat)
##
## Residuals:
##     Min      1Q  Median      3Q     Max
## -1.2044 -0.2272 -0.0385  0.2182  1.1778
##
## Coefficients:
##                                             Estimate Std. Error t value
## (Intercept)                                  1.54003    0.34931   4.409
## treatment1Irrigated                          3.15160    0.62166   5.070
## treatment2with bacteria                     -0.65534    0.36552  -1.793
## assim3                                      -0.36858    0.08211  -4.489
## treatment1Irrigated:treatment2with bacteria  1.93527    0.53733   3.602
##                                             Pr(>|t|)
## (Intercept)                                 0.000302 ***
## treatment1Irrigated                         6.81e-05 ***
## treatment2with bacteria                     0.088925 .
## assim3                                      0.000252 ***
## treatment1Irrigated:treatment2with bacteria 0.001901 **
## ---
## Signif. codes:  0 '***' 0.001 '**' 0.01 '*' 0.05 '.' 0.1 ' ' 1
##
## Residual standard error: 0.5952 on 19 degrees of freedom
## Multiple R-squared:  0.7379, Adjusted R-squared:  0.6828
## F-statistic: 13.37 on 4 and 19 DF,  p-value: 2.391e-05
```

```
emmeans(m3, pairwise ~ treatment1 * treatment2)
```

```
## $emmeans
##  treatment1 treatment2    emmean    SE df lower.CL upper.CL
##  Drought    w/o bacteria  -0.727 0.352 19    -1.46  0.00894
##  Irrigated  w/o bacteria   2.425 0.359 19     1.67  3.17575
##  Drought    with bacteria -1.382 0.450 19    -2.32 -0.44049
##  Irrigated  with bacteria  3.705 0.442 19     2.78  4.62889
##
## Confidence level used: 0.95
##
## $contrasts
##  contrast                                           estimate    SE df t.ratio
##  (Drought w/o bacteria) - (Irrigated w/o bacteria)    -3.152 0.622 19  -5.070
##  (Drought w/o bacteria) - Drought with bacteria        0.655 0.366 19   1.793
##  (Drought w/o bacteria) - Irrigated with bacteria     -4.432 0.711 19  -6.231
##  (Irrigated w/o bacteria) - Drought with bacteria      3.807 0.729 19   5.224
##  (Irrigated w/o bacteria) - Irrigated with bacteria   -1.280 0.359 19  -3.563
##  Drought with bacteria - Irrigated with bacteria      -5.087 0.823 19  -6.184
##  p.value
##   0.0004
##   0.3071
##   <.0001
##   0.0003
##   0.0102
##   <.0001
##
## P value adjustment: tukey method for comparing a family of 4 estimates
```

Assimilation by irrigation

```
ggplot(data=dat, aes(x=treatment1, y=assim3)) +
  geom_boxplot() +
  geom_point()
```

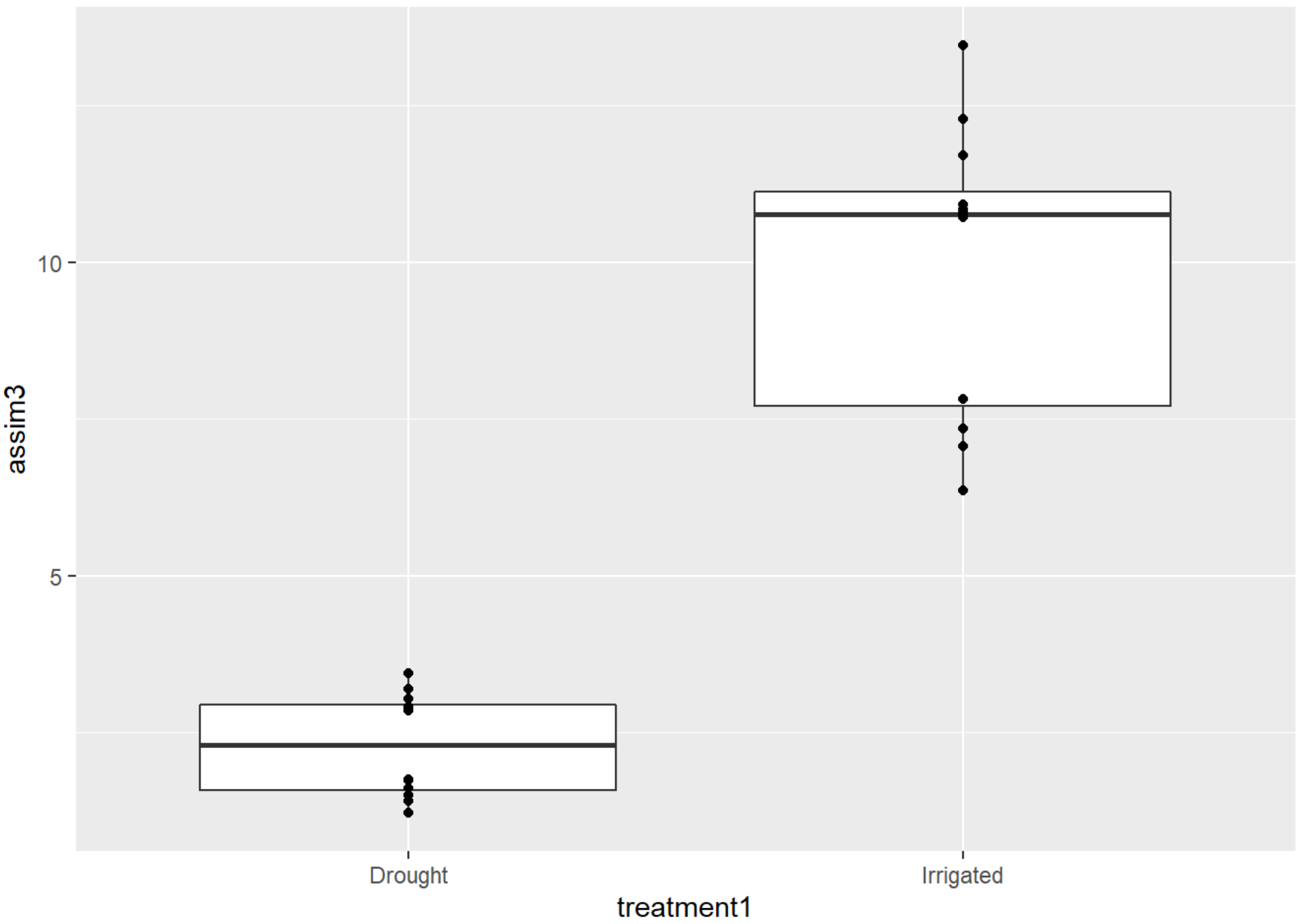


```
t.test(assim3 ~ treatment1, data=dat)
```

```
##
##  Welch Two Sample t-test
##
## data:  assim3 by treatment1
## t = -11.038, df = 13.804, p-value = 3.125e-08
## alternative hypothesis: true difference in means between group Drought and grou
p Irrigated is not equal to 0
## 95 percent confidence interval:
##  -9.204549 -6.206186
## sample estimates:
##   mean in group Drought mean in group Irrigated
##                2.297496               10.002864
```
